# A Case of Epidemic Cerebro-Spinal Meningitis

**Published:** 1895-02

**Authors:** W. Jarvis Barlow

**Affiliations:** New York, House Physician Mount Sinai Hospital


### A CASE OF EPIDEMIC CEREBRO-SPINAL MENINGITIS.

CURED

BY W. JARVIS BARLOW, M. D., NEW YORK,

House Physician Mount Sinai Hospital.

J. B., aged fourteen years, newsboy, was admitted to Mount Sinai Hospital on July 10, 1894, in the service of Dr. Manges, with whose kind permission the case is reported.

Family history negative. Parents could remember no previous sickness; always a strong and healthy child. The day before admission the boy was suddenly attacked with headache, vomiting and prostration. No history of traumatism. He vomited often during the day, and had much pain in the head, abdomen, and limbs. In the evening he became slightly delirious, and the parents noticed a slight rigidity of the head.

On admission the patient was unconscious; temperature, 102 degrees F.; pulse, 110; respiration, 26; face flushed; eyes brilliant and fixed; head rigid and slightly retracted; tenderness and stiffness of muscles of neck.

Examination of heart and lungs negative; abdomen tender, but no retraction; liver and spleen negative; patellar reflexes increased; movement of all extremities good. Soon restlessness and delirium became more marked, and with difficulty could only temporarily be controlled. The following day the symptoms were all severer. Increased retraction of head and stiffness of neck, no movement now being possible; marked tenderness over occiput and along the whole cervical region of the spine. Pupils react well; no strabismus or nystagmus. Abdomen extremely tender, with well-marked *taches cerebrales.* Patellar reflexes and all the skin reflexes increased. No ankle clonus.

*First Week.*—The fever followed an irregular course; lowest temperature, 100 degrees F.; highest, 104 degrees. Pulse at first rapid, then slow. Photophobia and intense hyperæsthesia; alternating delirium and stupor; no nystagmus; irregular movements of extremities.

*Second Week.*—Temperature still irregular; normal at times. Pulse, 70, small and compressible. Reflexes of limbs varied much; increased at times, then diminished in one or both legs. Alternating delirium and stupor still marked. Pupils dilated, reacting well; fundus of eye normal. Herpes labialis well marked. Beginning emaciation. Stiffness and flexion of forearms and hands.

At the end of this week (July 23d) both lower extremities were rigid and in a state of flexion, more marked on the right side.

Urine always contained some albumen; casts, hyaline and granular, now appeared.

*Third Week.*—Temperature higher, 102 degrees to 104 degrees. Extreme rigidity and retraction of the head. Flexion and stiffness of all the extremities. Marked emaciation.

Photophobia and hyperæsthesia less marked.

Delirium giving place to stupor and coma.

For two or three days had difficulty in deglutition. Retention of urine and fæces.

*Fourth Week.*—Temperature lower, 99 degrees to 102 degrees. Pulse higher, 110 to 120. Involuntary urination; hyperæsthesia still marked; reflexes varying the same. Tongue dry, brown, and fissured. Coma deeper.

*Fifth Week.*—Temperature sub-normal; pulse small and weak; emaciation extreme; coma still marked.

Pupils react well; no stagmus; fundus of eye normal.

*Sixth Week.*—The patient began to show improvement; less stiffness of head and extremities; all can be moved without pain. Temperature normal and on August 14th the patient showed consciousness. Voluntary urination and defecation. All symptoms abated, and on August 16th, consciousness was complete. Contractures of extremities disappeared gradually. Appetite returned.

*Seventh Week.*—The urine contained considerable quantities of pus acid in reaction. The temperature rose for a few days—remittent type—and the boy suffered much from pain on the left side of abdomen. There was distinct resistance and tenderness over the region of the left kidney. Under treatment, however, all symptoms subsided, and in several more days pus disappeared from the urine. After this convalescence was uninterrupted.

*Ninth Week.*—The patient out of bed, and on September 9th was able to walk. All special senses normal. Tongue clean; no tremor. Intelligence complete. September 20th, discharged from hospital cured.

The treatment of the case consisted in the first two weeks of ergot in large doses, bichloride of mercury, iodoform, bromides, and stimulants. Morphine and hyoscine to control delirium. For the temperature, sponging the body freely, and in the second week a few plunge baths at a temperoture of 65 degrees, which subsequently were abandoned on account of the pain and discomfort. Later, tonic treatment and forced feeding.

A recovery so complete without impairment of intelligence or any of the special senses, after so protracted a course, with well-marked symptoms, it seems to me, makes the case worthy of publication. At no time was there any eruption, although it was carefully looked for. The interesting fact of the pyelitis on the left side seems difficult of explanation and no mention of such is made by authorities to which I have had access.

